# Occupational Exposure to Carcinogens and Occupational Epidemiological Cancer Studies in Iran: A Review

**DOI:** 10.3390/cancers13143581

**Published:** 2021-07-16

**Authors:** Bayan Hosseini, Amy L. Hall, Kazem Zendehdel, Hans Kromhout, Felix M. Onyije, Rahmatollah Moradzadeh, Maryam Zamanian, Joachim Schüz, Ann Olsson

**Affiliations:** 1 Environment and Lifestyle Epidemiology Branch, International Agency for Research on Cancer (IARC/WHO), CEDEX 08, 69372 Lyon, France; HosseiniB@students.iarc.fr (B.H.); OnyijeF@fellows.iarc.fr (F.M.O.); SchuzJ@iarc.fr (J.S.); 2 Cancer Research Center, Cancer Institute of Iran, Tehran University of Medical Sciences, Tehran 1419733141, Iran; kzendeh@sina.tums.ac.ir; 3 Government of Canada, Charlottetown, PE C1A 1N3, Canada; amy.hall2@canada.ca; 4 Institute for Risk Assessment Sciences, Utrecht University, 3584 CL Utrecht, The Netherlands; h.kromhout@uu.nl; 5 Department of Epidemiology, School of Public Health, Arak University of Medical Sciences, Arak 3819693345, Iran; moradzadehr@arakmu.ac.ir (R.M.); m.zamanian@arakmu.ac.ir (M.Z.)

**Keywords:** occupation, occupational exposures, exposure measurement, cancer, Iran

## Abstract

**Simple Summary:**

Occupational cancers can be prevented by eliminating hazardous substances or by reducing workers’ exposures. Characterizing the extent of exposure to carcinogens in workplaces and industries is a crucial first step to exposure control. Iran is one of the most industrialized countries in the Middle East, yet lacks an overview of the extent of exposure to carcinogens and comprehensive risk management. This review provides an overview of studies conducted to date and demonstrates the need for interdisciplinary collaboration to inform occupational research and exposure control in Iran and beyond.

**Abstract:**

Introduction: The extent of exposure to occupational carcinogens is not well characterized in Iran, and little is known about the burden of occupational cancer. Objectives: This study aimed to describe exposure to occupational carcinogens and occupational epidemiology studies in Iran. Methods: Relevant studies up to January 2021 in Iran were identified through three databases (PubMed, Web of Science, and Google Scholar). Results: Forty-nine publications from 2009 to 2020 (one cohort, 11 case-control, 34 exposure monitoring studies, and three cancer burden studies) were included. The exposure monitoring studies were conducted mainly in the petroleum industry, metal industry, manufacturing of electronics, manufacturing of plastics, construction industry, and service industry. A few of the case-control studies also reported increased risk of cancers in relation to work in those industries. Conclusions: Occupational cancer epidemiology in Iran is at an early stage. Both epidemiological and exposure monitoring studies are generally limited in size to provide robust evidence of occupational cancer risks. A coherent strategy to estimate the occupational cancer burden in Iran should start with conducting epidemiological studies along with systematic monitoring of occupational carcinogens for use in hazard control and research.

## 1. Introduction

Industries in Iran have undergone rapid growth in recent decades. The largest industrial sectors are petroleum industries, followed by the manufacturing of motor vehicles and the pharmaceutical industry, mining and quarrying especially copper and aluminum, and manufacturing of metals and rubber and plastics products [1]. Workers in these industries may be exposed to various known or suspected carcinogens such as BTEXs (benzene, toluene, ethylbenzene, and xylene), crystalline silica, and heavy metals. National efforts to reduce the burden of occupational cancers require evidence-based information on exposures to carcinogenic agents and their related cancer risks in Iranian workers [2]. 

Exposure assessment is a critical component of all epidemiological studies and can be particularly complex in studies of occupational cancers. The choice of method depends to a large extent on the study’s design, resources, and what data are available. Exposure assessment applied in epidemiological studies frequently comprises indirect methods, including self-reports of jobs and/or various job-related exposures, and subsequent assignment of exposures by case-by-case expert assessment or job exposure matrices. In industrial cohort studies, employment records of workers and long-term routine surveillance of exposures in the workplace may be applied to conduct objective, detailed, and quantitative exposure assessment. Quantitative exposure assessment methods include personal air sampling, stationary air sampling, and biomonitoring to represent individual exposures during a specific time period. These direct exposure assessment methods are likely to be the most precise methods but reflect current or very recent exposures, unless repeated regularly over a long time period [3,4]. 

To our knowledge the presence of occupational carcinogens in workplaces in Iran have not been well characterized, and very little is known about the burden of occupational cancer in Iran and other Middle East countries. This is important because not just materials and technologies but also workplace environments and practices, legislated exposure controls used in industries might be different and unique for this region, making them difficult or impossible to infer from studies conducted elsewhere. For instance, the Iranian government adopted laws prohibiting the use of asbestos in final products in 2000 and the import of white asbestos in 2012, but it is still used in some industries such as automobile manufacturing instead of changing to non-asbestos materials [5]. Other factors determining differences in occupational exposures include the content of respirable crystalline silica in ground construction materials and the standard and types of busses and trucks affecting the motor emissions, etc. [6].

The objective of this review was to identify and characterize epidemiological studies of occupational cancers and exposure monitoring studies with projection of cancer risk in Iran and describe how relevant exposures were assessed and assigned to workers in these studies. 

## 2. Materials and Methods

### 2.1. Literature Search and Inclusion/Exclusion Criteria

A literature search was conducted to identify publications related to occupational cancer in Iran through three bibliographic databases (PubMed, Web of Science, and Google Scholar) from 1973 to January 2021. Select keywords were searched through title, abstracts, and body text to identify relevant publications. 

The search queries (Tables S1 and S2) included keywords according to the PECOs statement (Population: workers, and Iran, Exposure: occupational carcinogenic exposures, Comparison: unexposed/exposed workers, Outcomes: cancer) [7]. The search strategy combined the above terms by Boolean search operations (AND, OR, NOT) and Mesh terms. 

We included full publications of epidemiological studies (cohort and case-control studies on occupational cancer) and exposure monitoring studies in which the authors projected the subsequent risk of cancer. Occupational agents were restricted to chemical agents that had been evaluated by the International Agency for Research on Cancer’s (IARC) Monograph Programme on the Identification of Carcinogenic Hazards to Humans irrespective of its IARC classification, i.e., Group 1 (carcinogenic to humans), Group 2A (probably carcinogenic to humans), Group 2B (possibly carcinogenic to humans), and Group 3 (not classifiable as to its carcinogenicity to humans). All articles had to be published in English or Farsi. 

### 2.2. Data Extraction

Identified articles were imported into EndNote reference manager, and duplicates were removed. Two reviewers (B.H. & F.M.O.) independently screened the articles according to the PRISMA (Preferred Reporting Items for Systematic Reviews and Meta-Analyses) guidelines [8]. Titles and abstracts of all identified references in the primary search were screened to determine potential eligibility. In any case of disagreement between reviewers, the full-text publication was reviewed and discussed to resolve discrepancy; a third reviewer (A.O.) was consulted to reach consensus if needed. Following the primary screening, full texts of the relevant references were obtained. Five full-text articles had to be requested from authors via E-mail or ResearchGate, and all were successful. 

The following data were extracted from each retained publication: name of author, publication year, geographical region of Iran (North/South/West/East/Centre/Tehran), study design (Cohort/Case-control/Exposure monitoring with cancer risk projection as an outcome/burden of cancer studies with nonspecific study design, e.g., cancer projection using available data on exposure and outcomes), occupation (Title), industry (Title), exposure agents (Names), population size/sample size (Number of workers/samples), exposure assessment methods (Personal air monitoring/Biological monitoring/Stationary air monitoring/Self-report of job or exposure history/Registries/Expert Assessment), and study outcomes (Overall and specific cancer sites). 

## 3. Results

**Figure 1:**
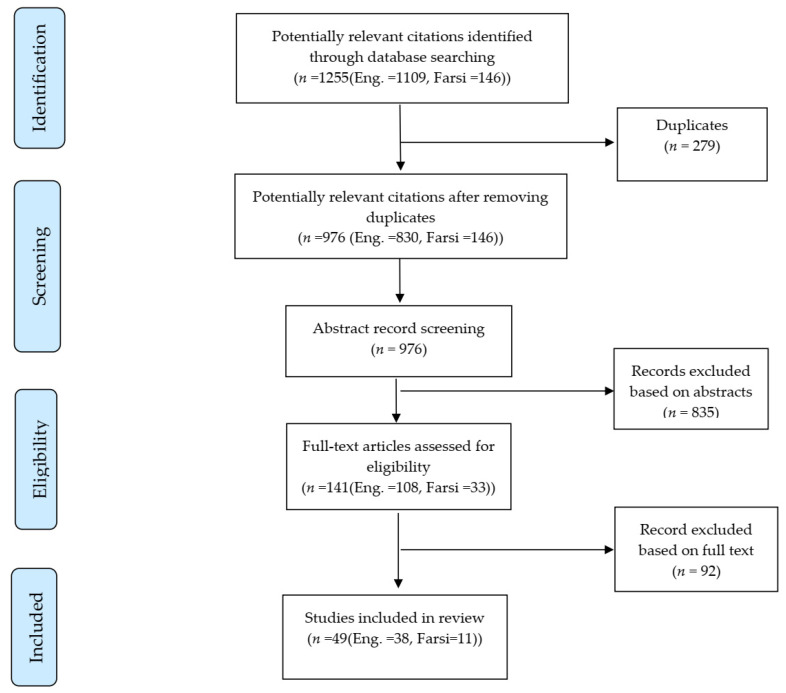
PRISMA flow diagram.

The publication selection process is summarized in the modified PRISMA flow diagram in Figure 1. The initial systematic searches identified 1255 publications (718 from PubMed, 391 from Web of Science, 146 from Google scholar). After removing the duplicates (*n* = 279), 976 publications remained. Of these, 835 were excluded by screening the titles and abstracts (in vitro and in vivo, nutritional studies, editorial, case reports, reviews and systematic reviews, physical and psychosocial exposure in the workplaces). 

The remaining 141 full-text publications were reviewed, and 92 were additionally excluded, i.e., studies evaluating occupational exposures in relation to health outcomes other than cancer. 

In total, 49 relevant publications (one cohort study, 11 case-control studies, 34 exposure monitoring studies with cancer risk projection as an outcome, and three cancer burden studies using national and international data) were retained for data extraction. The frequency of exposure assessment methods by study design are shown in Table 1.

### 3.1. Epidemiological Studies

The retained epidemiological studies (one cohort study and 11 case-control studies) are summarized in Table 2.

The only eligible cohort study included a random selection of 15,162 military veterans from the Iran–Iraq war between 1984 and 1987, who were followed prospectively for up to 25 years, with a particular interest of investigating the association of single exposure to sulfur mustard in relation to cancer incidence. Almost half of the cohort (*n* = 7570) had been exposed to sulfur mustard (at least one exposure documented in medical records while they were present in the battlefield) during the war and the other half had not been exposed (e.g., injured veterans). Survivors were followed up yearly by medical doctors, via telephone interview to confirm cancer occurrence or death. The outcomes of interest included the incidence of all types of cancer. Verification of cancer was performed by pathological confirmation [9]. 

Out of the 11 retained case-control studies, five examined bladder and urinary tract cancers. The exposure assessment in all case control studies was based on self-reported job histories and self-reported specific chemical exposures. The largest case–control study was conducted by Khoubi et al. (2013) [10] in the Isfahan Province. Using lifetime occupational history, job titles were coded according to the International Standard Classification of Occupation from 2008 (ISCO-08), and risks were estimated for 22 pre-defined occupational groups. This study observed a significantly increased risk of bladder cancer in certain occupations, including truck and bus drivers, agricultural workers, metal industry workers, construction workers, and domestic housekeepers. 

Aminian et al. (2014) [11] conducted a hospital-based case-control study in men to evaluate the risk of bladder cancer in relation to occupational exposure. The authors stated, “controls were male cases without cancers and occupational exposures”. The results were presented for a priori assigned high and low cancer-risk jobs. The statistical analysis section comprised very limited information, and the study reported elevated crude odds ratios for jobs allocated to high-risk occupations including bus and truck drivers, road and asphalt workers, mechanics, refinery and petrochemical workers, plastic manufacturing, metal manufacturing, welding, and pipeline workers.

Farzaneh et al. (2017) [12] conducted a case-control study on bladder cancer in the Yazd Province. Job titles were classified into a priori “high-risk” and “low-risk” jobs based on their potential (according to the literature and expert’s opinion) for exposure to bladder carcinogens such as aromatic amines, amino-biphenyl, polycyclic aromatic hydrocarbons [PAH], and azo dyes. An elevated risk of bladder cancer was observed among a priori high-risk jobs including metal manufacturing, textile, driving, agriculture and livestock, and construction.

Ghadimi et al. (2015) [13] reported on a hospital-based case-control study on urinary bladder cancer in relation to occupation, smoking, and opium consumption, conducted in the Kurdistan Province. By the occupation classification ISCO-08, univariable analysis showed an elevated risk of bladder cancer in workers in metal manufacturing. 

Tajvidi et al. (2013) [14] conducted a case-control study of kidney cancer in the Isfahan Province. Study participants were asked about occupations held as well as selected exposures in a questionnaire. Results were reported based on Chi-square and T-test analysis, which compared the occupations and exposure difference between cases and controls. In case subjects the frequency of agriculture and laborers in various industries was higher than that in other occupations. Exposures to pesticides, paint, petrol, chemicals, and mineral agents were significantly more frequent in cases than in control subjects.

A small case-control study of head and neck cancer showed an association with pesticide exposure. Exposure to pesticides was confirmed by residual pesticide from the adipose tissue of the neck of cases and controls. Information on pesticide exposure was classified into non exposed, low, and high exposed based on occupational, residential, and agricultural activities history [17]. 

A study of malignant lymphoma conducted in the Fars Province observed an elevated risk of Non-Hodgkin lymphoma related to pesticide exposure. Information on all jobs held and exposures for each job, as well as detailed information of agricultural exposures was collected. For the analyses, job titles were classified by major groups of ISCO-68. The results showed elevated risk of lymphoma in farmers compared to all other jobs. The elevated risk was present for any type of pesticide exposure whether occupational or residential [16]. 

Another hospital-based case-control study of non-Hodgkin and Hodgkin’s lymphoma was conducted in the Tehran Province. In this study, job titles were classified into high- and low-risk jobs based on the literature. Elevated risks of non-Hodgkin lymphoma were observed in unadjusted analyses for occupations such as welders and metal workers, with increased risk of Hodgkin lymphoma in drivers [15].

Hosseini et al. (2009) [18] conducted a hospital-based case-control study to investigate the lung cancer risk related to known and suspected environmental and occupational lung carcinogens. In this study, increased risk of lung cancer was observed for occupational exposure to inorganic dusts, chemical compounds, and heavy metals. The same results were observed in smokers and nonsmokers. 

A hospital-based case-control study on gastrointestinal cancer among male patients was conducted in the Tehran Province. One-digit major groups of the ISCO-08 classification were analyzed. The results reported an elevated risk of gastrointestinal cancers in plant and machine operators, assemblers, agricultural workers, and laborers [19]. 

A case-control study of breast cancer conducted in the Mashhad Province recruited participants among employed women classified into four major groups: teachers, administrative/clerical workers, healthcare workers, and miscellaneous jobs (carpenters, hairdressers, tailors, cooks), and the results showed only an increased risk of breast cancer in teachers compare to the other occupations [20].

All case-control studies were relatively small, with a mean number of 180 cases (range 31–300) and 221 controls (range 32–500). The cases were mostly recruited from hospitals (*n* = 8) and in three publications from cancer registries. The controls were recruited from hospitals (*n* = 6), general population (*n* = 2), neighbors (*n* = 1), relatives of cases (*n* = 1), and unclear (*n* = 1). Most studies included only histologically confirmed cases (*n* = 8). The exposure assessment in all case-control studies was mostly based on self-reported jobs and/or exposures. Except two studies which allocated jobs to the low- vs high-risk jobs based on the literature review or expert judgement, no subsequent attempt was made to assign exposures to these jobs (e.g., expert assessment or existing job-exposure-matrices). Two publications considered specific exposure to pesticides, while the others addressed jobs and industries as proxies for occupational exposures. 

### 3.2. Exposure Monitoring Studies Including Cancer Risk Predictions

Table 3 describes characteristics of the 34 exposure monitoring studies that subsequently estimated cancer risk. Sixteen studies used personal air monitoring, 13 used stationary air monitoring, and three studies applied a combination of personal and stationary air monitoring to estimate the level of exposure of individual workers and workplaces. One additional study estimated the lung cancer risk based on available exposure measurement data from previous studies (used available input parameters, e.g., intensity of nickel and chromium exposure in welders from published studies) [21]. Another study calculated exposure using an exposure index (EI) based on the physical characteristics of the agent, e.g., the olfactory threshold value, inhalable fraction and vapor, the preventive measures including personal and environmental protection, level of benzene exposure, and work duration per week, and estimated the risk of leukemia [22]. 

The majority of exposure monitoring studies (14/16 of personal air sampling and 12/13 of stationary air sampling) measured the exposure of workers only once over an entire work shift, while two studies collected repeated measurements of the same worker on two or three occasions during an entire work shift [23,24]. One study measured exposure via personal air sampling over the full duration of a work shift for three consecutive months [25]; another assessed exposure by taking four short-term personal air measurements (90 min) in different hours of a work shift for five consecutive weeks [26].

In larger industries, exposures were assigned to workers based on their tasks in relevant production units [24,27,28,29]. In small workplaces, e.g., beauty salons [30,31], waterpipe cafés [32,33], and gas stations [34], stationary air monitoring was applied to estimate exposure during a work shift.

The most frequently monitored agents in the monitoring studies were volatile organic compounds (VOCs) including benzene, toluene, ethylbenzene, and xylenes (BTEXs). Personal and stationary air sampling of these agents was conducted in a variety of industries, e.g., petroleum industry [23,27,35], manufacture of basic iron and steel [36], automobile manufacturing [37], painting units of automobile manufacturing [38], poultry slaughterhouse [26], and gasoline and Compressed Natural Gas (CNG) refueling stations [39]. 

In exposure monitoring studies, monitoring results were used to project the risk of cancer using different terms, e.g., Excess Lifetime Cancer Risk (ELCR), Incremental Lifetime Cancer Risk (ILCR), Inhalation Lifetime Cancer Risk (LTCR), and Lifetime Cancer Risk (LCR). 

In water pipe cafés [32], and beauty salons [30,31], the investigators used stationary air monitoring exclusively to measure the exposure to BTEXs and PAHs. Exposures were monitored with consideration of sampling sites, e.g., type of ventilation system and the location at which those salons or cafés were located, e.g., ground floor or basement. 

Crystalline silica was the second most frequently monitored agent. Workers’ exposure was measured by personal and stationary air sampling in a variety of industries such as casting of iron and steel manufacture [40], manufacture of ceramic pin insulators [41], construction workers at building demolition sites [42,43], and in sectors including stone cutting and milling, foundries, glass manufacturing, asphalt manufacturing, construction, sand and gravel mining, sand blasting, and ceramics, bricks and cement manufacturing [44]. Based on the monitoring results, the risk of lung cancer was estimated. 

In a few studies, exposure to formaldehyde was monitored in pathology laboratory staff through personal and stationary air sampling [45,46,47], and in one study exposure to formaldehyde was assessed by personal air sampling of plastic product workers to estimate their cancer risk [48]. 

Exposure to vinyl chloride was measured among plastic manufacturing workers through personal air samplers to estimate the ELCR [49].

**Table 3:** Characteristics of studies with exposure monitoring design.

Reference/Location/Study Year	Industry	Population/Sample Size/Description	Exposure Agents Assessed	Type of Assessment/Frequency of Measurement	Outcome/Results
Heibati/North of Iran/2016 [27]	Petroleum	50 workers selected randomly (tanker loading workers, tank-gauging workers, drivers, firefighters, and office workers)	BTEXs ^1^ (benzene, toluene, ethylbenzene, and xylene)	Personal air sampling; over one 8-h work shift	Elevated ELCR ^2^ in tanker loading and tank-gauging workers attributable to tasks, e.g., dispensing, loading, and unloading of petrol
Shanh/Iran/2013–2014 [23]	Petroleum	338 samples from 169 workers in 21 petrochemical complexes including mechanics, security, samplers, site men, technicians, laboratory staff, and office workers	VOCs ^3^ (benzene, epiclorohydrine, trichloroethylene, styrene, ethyl benzene, and 1,3-butadiene)	Personal air sampling; two samples taken over a work shift for each subject; each sample duration 3.5–4 h	Elevated LCR ^4^ for all workers attributed to VOC carcinogenic components; the highest LCR was attributable to benzene exposure
Sadeghi-Yarandi/Isfahan, Iran/2018 [24]	Petroleum	150 samples from 50 workers including fire-fighters, polybutadiene latex (PBL), dryer, and coagulation workers, mechanics, electricians, packing, and laboratory workers	1,3-butadiene	Personal air sampling; 90 min for each sample (beginning, middle, and end of work shift)	Elevated LCR for all workers, highest LCR attributable to 1,3-butadiene observed in safety and fire-fighters of petroleum complexes
Barkhordari/Iran/2013 [21]	Petroleum	30 welders were assigned to different groups by exposure to Ni and Cr (VI) and also health situation (healthy and asthmatic welders)	Nickel and hexavalent chromium from welding fumes	Personal air measurement data; parameters (e.g., intensity and duration of exposure) were used from previous studies; information on daily work hours, daily tasks collected by questionnaires	Elevated ILCR ^5^ in exposed group in both asthmatic and healthy welders
Golbabaie/Iran/study year is unknown [22]	Petrochemical	Not applicable	Benzene	Exposure data used from previous prospective study in rubber industry workers; exposure index (EI) determined by experts using physical characteristics of exposure agent (e.g., the olfactory threshold value, inhalable fraction and vapor), and preventive measures including personal and environmental protection.	Elevated risk of leukemia attributable to benzene exposure in all workers
Harati/Iran/2016 [35]	Petrochemical	123 samples from 60 workers	VOCs (benzene, toluene, xylene, pentane, hexane, heptane, octane, and nonane), and hydrogen sulfide (H_2_S)	Personal air sampling; two samples from each worker (one to measure VOCs, another to measure H_2_S), and 3 control samples; details on sampling duration were not provided	Elevated ELCR in workers attributable to benzene exposure in all workers
Hazrati/Ardabil, Iran/study year is unknown [39]	Gasoline and CNG ^6^ refueling stations	Samples from 24 refueling stations including 15 petrol and 9 CNG stations; the population sample size was not clarified	BTEXs	Personal air sampling over a full work shift	Elevated long-term exposure cancer risk related to BTEX compound exposure
Javadi/Isfahan, Iran/2016 [34]	Gasoline and CNG refueling stations	24 samples from 12 refueling stations (12 workers, 12 stationary air sampling)	BTEXs	Personal air sampling and stationary air sampling over a full work shift	Elevated risk of cancer attributable to benzene exposure
Omidi/Tehran, Iran/2018 [26]	Poultry slaughterhouse	200 samples and 40 blank samples from all workers	VOCs (benzene, toluene, ethylbenzene), and CS2 (Carbon disulphide)	Personal air sampling; 4 samples in a different hours of a full work shift; sampling repeated in 5 consecutive weeks for each worker; each sampling duration was between 50 and 90 min	Elevated LCR attributable to benzene exposure
Mohammadyan/Neyshabur, Iran/2017–2018 [50]	Electronic manufacturing, plastic compress unit	141 samples from 59 workers including primary granule warehouse, plastic injection workers, shift managers, miscellaneous (forklift drivers, quality control engineers, and crew)	Styrene	Personal air sampling, details on sampling duration were not provided	Elevated LCR attributable to styrene exposure in plastic injection operators and shift supervisors
Mohammadyan/Neyshabur, Iran/2017–2018 [28]	Electronics manufacturing	40 samples from 40 female soldering workers (cutting electrical wires and coating, initial soldering, voltage testing and secondary soldering workers, and shift supervisors)	Lead	Personal air sampling over a full work shift	ELCR not increased in high-risk exposed group
Tayfeh Rahimian/Tehran, Iran/study year is unknown [49]	Manufacturing of plastics products	100 workers from mixing, winder, coating, finishing, rewinder, warehouse used to manufacture two plastic products	Chloride vinyl	Personal air sampling over a full work shift	Elevated ELCR attributable to duration of chloride vinyl exposure
Mazinani/Tehran, Iran/study year is unknown [48]	Manufacturing of plastics products	54 samples from 4 workshops in different units including cutting, stoning, pressing, and packing	Formaldehyde	Stationary air sampling over a full work shift. Indoor air temperature, pressure, and relative humidity used in analyses	Elevated risk of cancer attributable to formaldehyde exposure in all units; the highest level of exposure was in stoning and pressing units
Azari/Iran/study year is unknown [25]	Manufacturing of shoes	Sample from 48 workers in 12 workshops including cutting, modelling, fitting, and finishing tasks	Benzene, toluene	Personal air sampling, once a month during 3 consecutive months, details on sampling duration were not provided	Elevated risk of leukemia attributable to benzene and toluene exposure in shoemakers; the level of exposure was higher than the threshold limit value (TLV)
Sanjari/Iran/study year is unknown [51]	Aluminum rolling	103 samples from different units including slabbing, production, painting, and washing workshops	Chemical exposure including aluminum products, silica, rock wool, iron oxide, manganese, sulfuric acid, benzene, ethylbenzene, toluene and xylene	Stationary air sampling; details on sampling duration were not provided; indoor air temperature, pressure, and humidity used in analyses	Elevated ELCR attributable to benzene exposure; level of exposure to sulfuric acid in washing lines and manganese in the manufacturing unit were higher than in other units
Zarei/Tehran, Iran/2017 [40]	Foundry	Sampling of breathing zone of 55 workers from different units including machine operator, painting, furnace, and cleaning; information on number of samples was not provided	Crystalline silica	Stationary air sampling, 4 h during a work shift	Elevated lung ELCR attributable to crystalline silica exposure; All workers exposed to a higher level than the definite acceptable limit recommended by OSHA ^7^
Omidianidost/Tehran, Iran/2011 [52]	Foundry	80 samples of 80 workers from workshops used for 29 small foundries including 10 iron cast, 3 brass, and an aluminum foundry	Crystalline silica	Personal air sampling over a full work shift	Elevated risk of lung cancer mortality; 50 percent of workers exposed to greater than the acceptable threshold limit value by OSHA
Omidi/Iran/study year is unknown [36]	Steel manufacturing	50 samples from the breathing zone of 372 workers in energy and biochemistry units, benzol refinement and experimental furnace units	BTEXs	Stationary air sampling over a full work shift	Elevated cancer risk attributable to benzene exposure; exposure to BTEX components in the benzol refinement unit was higher than in other units
Normohammadi/Tehran, Iran/2010–2011 [42]	Construction	60 demolition workers from 4 demolition sites; 15 samples from each site	Crystalline silica	Personal air sampling over a full work shift, meteorological parameters including air temperature and wind speed were used in the analysis	Elevated ELCR of lung cancer attributable to crystalline silica exposure in workers
Tavakol/Tehran, Iran/study year is unknown [43]	Construction	85 samples from 85 construction workers including supervisors, stonemason, batching and concrete workers, and labors	Crystalline silica	Personal air sampling over a full work shift	Elevated ELCR of lung cancer attributable to crystalline silica exposure in construction work; batching and concrete workers had the highest average exposure that was greater than the threshold limit recommended by OSHA
Moghadam/Neyshabour, Iran/2015 [53]	Concrete manufacturing	Sampling of breathing zone of 72 workers from autoclave units, wing tube and cutter line, mixing, packing, and quality control units; information on number of samples was not provided	Crystalline silica	Stationary air sampling; information on sampling duration was not provided	Elevated ELCR for lung cancer attributable to crystalline silica exposure
Azari/Tehran, Iran/study year is unknown [44]	Stone cutting and milling, foundry work, glass manufacturing, asphalt, construction, sand and gravel mining, sand blast, ceramics, bricks and cement	200 workers from 50 workplaces (4 workers in each) including stone cutting and milling, foundry, glass manufacturing, asphalt preparation, ceramic, brick and concrete manufacturing, and construction	Crystalline silica	Personal air sampling over a full work shift	Elevated lung ELCR attributable to cumulative exposure of crystalline silica (direct correlation between the level of exposure and lung cancer risk)
Mohammadi/Markazi, Iran/2015 [41]	Ceramic pin insulator manufacturing	60 samples from 5 units including pressing, production, coating, furnace, and packing (12 samples in each unit)	Crystalline silica	Stationary air sampling; information on sampling duration was not provided	The highest mortality risk of lung cancer attributable to crystalline silica exposure was estimated for furnace workers
Yahyaei/Rasht, Iran/2018 [47]	Hospital	65 employees in pathology labs including pathologist, lab technician, office worker, service workers	Formaldehyde	Personal air sampling during direct exposure to formaldehyde (8:00 and 12:00 a.m.); 25 min for each task; stationary air sampling during a full work shift	Elevated individual lifetime cancer risk 100–1000 times higher than the acceptable cancer risk in all exposed laboratory staff; exposure level in all staff members was higher than the acceptable level by OSHA
Pourtaghi/Tehran, Iran/2018 [46]	Hospital	68 samples from the breathing zone of 72 hospital staff	Formaldehyde	Stationary air sampling during one full work shift	Elevated LCR attributable to intensity of formaldehyde exposure greater than the recommended acceptable limit by OSHA
Jalali/Iran/2019 [45]	Hospital	Sampling of breathing zone of 60 pathology laboratory staff members; information on number of samples was not provided	Formaldehyde	Stationary air sampling during one full work shift	Highest LCR attributable to formaldehyde in lab technicians
Zarei/Tehran/Iran/2010 [29]	Brake shoe and clutch disk manufacturing	61 workers including weighing, mixing, pressing, and finishing occupations	Asbestos	Personal air sampling; 4 h over a full work shift	Elevated risk of lung cancer ELCR attributable to cumulative exposure of asbestos; exposure levels for all workers far greater than the occupational exposure limits recommended by OSHA
Jafari/Isfahan, Iran/study year is unknown [54]	Asbestos-cement products manufacturing	97 workers from 4 units including milling, cutting, and cutting	Asbestos	Personal air sampling; samples collected from different units of the factory over a full work shift	Elevated risk of lung, mesothelioma, and gastrointestinal cancer mortality after 20 years of exposure; greater risk in the dry cutting unit than the wet cutting unit
Harati/Iran/2015 [37]	Automobile manufacturing	46 samples from 20 workers	BTEXs, Silica	Personal air sampling (2 times for each worker over a full work shift), and 6 stationary air samples over a full work shift	Elevated hematological cancer risk corresponding to cumulative exposure to benzene and crystalline silica
Dehghani/Iran/2016 [38]	Automobile manufacturing	34 samples from breathing zones of workers: cabin maker, pre-paint, and painting units	BTEXs	Stationary air sampling over a full work shift	Elevated risk of cancer in painting unit workers attributable to benzene and ethyl benzene with 30 years of exposure
Baghani/Ardabil, Iran/2017 [31]	Beauty salons	50 beauty salons across the Ardabil Province	BTEXs	Stationary air sampling; indoor air temperature, pressure, and relative humidity were used in the analysis, samples taken during the afternoon (14:00 to 19:00)	Elevated LTCR ^8^ attributable to BTEX components
Hadei/Tehran, Iran/2016–2017 [30]	Beauty salons	360 samples from 20 beauty salons, 180 samples each for indoor and outdoor; (3 samples for each component including BTEXs, formaldehyde, and acetaldehyde)	BTEXs, formaldehyde, and acetaldehyde	Stationary air sampling; over a full work shift during 3 consecutive months; air temperature, pressure, and relative humidity were used in the analysis	Elevated risk of cancer attributable to benzene, formaldehyde, and acetaldehyde exposure; exposure to different components affected by tasks: benzene and toluene (hair dying), formaldehyde (hair style and nail treatments), and xylene (hair styling)
Hazrati/Ardabil, Iran/study year is unknown [32]	Waterpipe café	87 samples from 81 waterpipe cafés	BTEXs	Stationary air sampling; each sample duration lased for 50 min; sampling from 14:00 to 19:00; 6 additional samples taken directly from the smoke mainstream of the waterpipe (4 from fruit flavored tobacco and 2 from regular tobacco).	Elevated long-term exposure cancer risk attributable to benzene exposure
Rostami/Ardabil, Iran/2018 [33]	Waterpipe café	51 samples from 51 waterpipe cafés	PAHs ^9^	Stationary air sampling; from breathing zone of smokers and employees; air temperature, pressure, and relative humidity were used in the analysis	Elevated risk of cancer attributable to PAH component inhalation

^1^ Benzene, Toluene, Ethylbenzene and Xylenes; ^2^ Excess Lifetime Cancer Risk; ^3^ Volatile Organic Compound; ^4^ Lifetime Cancer Risk; ^5^ Incremental Lifetime Cancer Risk; ^6^ Compressed Natural Gas; ^7^ Occupational Safety and Health Administration; ^8^ Inhalation Lifetime Cancer Risk; ^9^ Polycyclic Aromatic Hydrocarbons (PAH), 16 PAH compounds including Naphthalene (Naph), Acenaphthylene (Acy), Acenaphthene (Ace), Fluorene (Flu), Phenanthrene (Phen), Anthracene (Anth), Fluoranthene (Flt), Pyrene (Pyr), Benzo[a]anthracene (BaA), Chrysene (Chr), Benzo[b]fluoroanthene (BbF), Benzo[k]fluoroanthene (BkF), Benzo[a]pyrene (BaP), Dibenzo[a,h]anthracene (DahA), Benzo [ghi]perylene (BghiP), and Indeno[123-cd]pyrene (Ind).

### 3.3. Burden of Cancer Studies

In addition, there were three relevant publications that used secondary data to project occupational cancer risk [55,56,57]. Of these, two publications by Mosavi-Jarrahi et al. projected the fraction of leukemia and lung cancer incidence in Iran attributable to exposure to occupational carcinogens, e.g., benzene, ionizing radiation, and ethylene oxide for leukemia, and silica, cadmium, nickel, arsenic, chromium, diesel fumes, beryllium, and asbestos for lung cancer [55,56]. Both publications applied available country-specific workforce survey data from the International Labour Organization (ILO) and estimated the proportion of exposed workers in each industry using the European CARcinogen Exposure (CAREX) database and relative risk estimates from the literature to predict the burden of occupational cancer [58]. The authors reported that 0.08% of male workers and 0.02% of female workers were exposed to the major occupational lung carcinogens (silica, cadmium, nickel, arsenic, chromium, diesel fumes, beryllium, and asbestos), resulting in an attributable fraction of lung cancer due to occupational exposures of 12% in men and 1.5% in women [55], and that 0.016% of male workers and 0.02% of female workers were exposed to leukemogens (benzene, ionizing radiation, and ethylene oxide), estimating that 7.6% of leukemia in men and 3.6% in women were attributable to occupational exposures [56].

Abtahi et al. [57], estimated age-, sex-, and cause-specific mortality attributable to occupational risks for the years 1990, 2005, and 2015 using the methods provided in the Global Burden of Disease project (GBD) 2015 [57]. The occupational carcinogens included in the projections were asbestos, arsenic, benzene, beryllium, cadmium, chromium, diesel exhaust engine, formaldehyde, nickel, polycyclic aromatic hydrocarbons, second-hand smoke, silica, and sulfuric acid. Information on industry patterns was extracted from National Population and Housing censuses in 1986, 1996, 2006, 2011, and 2016, and exposures by industry were derived primarily from the European CAREX database [59,60]. The results of this study showed that exposure to particulate matter, gases, and fumes, and asbestos was among the highest contributions to the national attributable DALYs in 2015 among occupational risks. In addition, from 1990 to 2015, the increase in total DALYs attributable to occupational carcinogens (112%) was higher than that for other occupational risks.

### 3.4. Publications over Time and Location

There were no relevant publications before 2009; in the subsequent years, the trend fluctuated between two to four publications per year until 2015. In 2016 to 2021, the trend was between five to seven publications per year. Only a few epidemiological studies per year were published between 2009 and 2021. Figure S1 provides a full summary of the time trends.

In terms of the geographical trend of publications, the greatest number (*n* = 15 or 31%) were at the national level or in non-specified areas of Iran in various manufacturing industries. The second most frequent geographical area was Tehran 11 (22%), the capital of Iran with a population of around 9 million, including multiple types of economic activities. Studies in Tehran were conducted across diverse workplaces such as manufacturing of plastic products, manufacturing of parts and accessories for motor vehicles, hairdressing and other beauty treatment, hospitals, and the construction industry. Studies conducted in the central part of Iran (Isfahan, Arak, Yazd, and Kashan; *n* = 8) included mostly heavy industries such as casting of iron and steel (*n* = 2), the manufacture of basic metals including iron and steel, and plastic products (*n* = 6) (Figure S1).

## 4. Discussion

This review identified 49 articles on occupational exposure to carcinogens and associated cancer risk in Iran. The studies consisted of one cohort study and 11 case-control studies directly investigating the association between occupational exposures and the risk of various cancers, as well as 34 studies with exposure monitoring including projections of associated cancer risks, and three studies investigating the burden of cancer related to occupational carcinogen exposure based on secondary national and international survey data.

The first relevant article published in 2009 indicates that occupational cancer epidemiology is a relatively new topic in Iran, with the number of publications increasing in recent years. The relatively low number of published articles on occupational cancers overall may be due to several reasons including (1) occupational physicians and hygienists focusing on acute effects of occupational exposure rather than cancers that take many years to develop, (2) limited tradition of collaboration across disciplines, e.g., hospitals, cancer registries, universities, and relevant ministries, (3) it may be that some research is conducted but not published in national and international journals, and (4) a lack of training and capacity in this particular area of research.

Even though the petroleum industry is a major industry in Iran [1], only six publications focused on this industry. This could be due to confidential matter and safety precautions in this industry that would require permission and special security training for external researchers [61].

Regarding the epidemiological studies, the only identified prospective cohort study with a long-term follow-up examined cancer incidence in relation to a single exposure to sulfur mustard in military service personnel in the Iran–Iraq war (which has been evaluated by IARC monograph as an occupational exposure agent with sufficient evidence for lung cancer) [62]. This cohort study also included volunteers who joined the armed forces temporally to defend their cities with unknown job histories, so it is not a classical occupational cohort with precise exposure information.

In case–control studies, occupational information was collected via interviews, by self-reporting of jobs and/or occupational exposures, with no further attempt to assign occupational exposures. Some of the case–control studies featured design aspects (e.g., choice of controls, potential confounding, and power) that will have led to limitations in the interpretation of the results. One of the case–control studies appeared to have selected controls in an inappropriate way (by selecting them based on the absence of exposure), but could be related to authors’ uncertainty about the study design or mistranslation from Farsi to English in the English publication [11].

This review found that exposure monitoring studies in Iran mostly focused on the projection of cancer risk based on a limited number of exposure measurements (personal and stationary air sampling). Thirteen out of 34 studies assessed the exposure through stationary measurements that provide only a very crude indication of personal exposure. Moreover, exposure agents such as BTEXs, crystalline silica, formaldehyde, and chromium in the workplaces were mostly measured on single occasions, although it is well known that exposure levels often differ between and within workers form day-to-day and are not constant over time (months, seasons, and years) [63,64]. Estimating the risk of cancer based on the exposure level derived from a single (personal) exposure measurement may over- or underestimate risk as this value may not be representative for the individual’s exposure over time [63]. A few of the studies used previously established exposure databases, e.g., CAREX to project the occupational cancer burden [58]. CAREX is a database which contains estimates of numbers of workers occupationally exposed to carcinogens by European industries (exposure data from 1990–1993). As shown by the two studies applying CAREX and GBD data to estimate the occupational cancer burden in Iran, the burden appears relevant, but these estimations have large uncertainties and do not provide insight into targeted prevention measures, as they are based on extrapolating exposure from other countries, workforce, and risk data instead of structured representative (exposure and risk) data from Iran.

This review provides the first overview of the status of occupational cancer research in Iran. A major limitation of the review is that carcinogenic exposures summarized in non-cancer studies are not captured here. Further, studies that have not been published in journals indexed by the searched databases might have been missed. Some studies were not sufficiently detailed to retrieve all relevant information, e.g., “questionnaires assessed occupational exposures” but did not indicate the type of exposure.

## 5. Conclusions

Identifying the burden of occupational cancers is an important step to identify the extent of a problem and develop prevention measures to reduce risks [2]. Since industrial and regulatory circumstances vary across countries, well-designed occupational cancer studies of sufficient size that include relevant exposure assessment methods for each study design are needed in Iran. Enhanced collaboration, between, e.g., occupational physicians, cancer researchers, industrial hygienists, and workplace representatives, as well as collaborations and participation in international conferences [65], would be an asset to conduct informative studies.

Additionally, further systematic exposure monitoring surveys will help to identify potential high-risk occupational environments for future risk management planning and facilitate and can inform the exposure assessment and assignment of future epidemiological studies [64,66].

We recommend that further work be focused in three key areas. First, given that there are large-scale ongoing cancer epidemiology studies in Iran (for example, the Persian cohort study [67] and the IROPICAN case-control study [68]), proper occupational exposure assessment and assignment should be included in these studies, which requires the development of valid job-exposure matrices specifically for Iran. Second, large-scale cohort studies in major industries should be initiated to inform cancer control in Iran and efforts on a global scale, as data from emerging economies are lacking worldwide [69]. Third, occupational exposure monitoring needs to be more systematic, with data made (centrally) available for (epidemiological) research and to develop risk reduction measures.

All these efforts will support the ultimate goal of occupational cancer prevention in Iran. Further, the documentation of exposure levels, contexts, and cancer risks in Iran will contribute to the scientific understanding of occupational cancers and related exposure factors on a global scale.

## Figures and Tables

**Table 1:** Relative frequencies of exposure assessment methods and study outcomes by study design.

Study Characteristics	Cohort *n* = 1 (%)	Case-Control *n* = 11 (%)	Exposure Monitoring *n* = 34 (%)	Cancer Burden Studies *n* = 3 (%)
Direct exposure assessments	-	0 (0.0)	32 (94.1)	-
Personal Air Sampling	-	-	16 (47.0)	-
Stationary Air Monitoring	-	-	13(38.2)	-
Stationary/Personal Air Sampling	-	-	3(8.8)	-
Indirect Exposure Assessment Methods:	1 (100)	11 (100.0)	2 (5.8)	3 (100)
Self-report of job or exposure history	-	11 (100.0)	-	-
Available databases	-	-	1 (2.9)	3 (100)
Expert Assessment	-	-	1 (2.9)	-
Not applicable	1 (100)	-	-	-
Cancer Outcomes				
Cancer, general ^1^	1 (100)	-	24 (70.5)	1 (33.3)
Lung cancer	-	1 (9.0)	8 (24.3)	-
Bladder and urinary tract cancer	-	5 (45.4)	-	1 (33.3)
Hematological cancer	-	2 (18.1)	2 (8.1)	1 (33.3)
Head and neck cancer	-	1 (9.0)	-	-
Breast cancer	-	1 (9.0)	-	-
Gastrointestinal cancer	-	1 (9.0)	-	-

^1^ General term representing lifetime cancer risk, long-term exposure cancer risk, risk of any cancer, etc.

**Table 2:** Characteristics of epidemiological studies.

Reference/Location/Study Year	Cancer Sites	Population Size/Description	Source of Exposure Assessment	Exposure Duration Measure	Covariates Controlled for in Modelling	Outcome (Risk of Cancer)
Prospective cohort						
Zafarghandi/Iran/1984–2010 [9]	Cancer	7570; Male veterans, Exposed to sulfur mustard during the Iran–Iraq war (1984–1987); 7592; Male veterans, unexposed to sulfur mustard (instead, e.g., physical injuries).	Documented medical records on at least one acute exposure while the participant presented in the battlefield between 1984 and 1987	Single exposure to sulfur mustard during the war	Stratified by age, smoking status, educational level, marital status	Yes
Case-control						
Khoubi/Isfahan, Iran/2004–2009 [10]	Bladder	Cases: 300; bladder cancer patient recruited from registry Controls: 500; population-based controls	Questionnaire; Phone/face-to-face interview; Collected data on industry title, duration, hours of work per day, self-report of exposures	Lifetime occupation history: Jobs held > 6 months	Age, sex, smoking status (never/current/former smoker, duration of smoking (year), average number of cigarettes per day)	Yes
Aminian/Tehran, Iran/2007–2009 [11]	Bladder	Cases: 160; Male- Histologically confirmed Controls: 160 Male without occupational exposure to the chemicals (source of controls recruitment is unclear)	Study specific questionnaire; Face-to-face interview; Collected data on job title, duration, exposure to the specific chemicals in each job, history of cancer occurrence in coworkers	Current and former jobs	Sex	Yes
Farzaneh/Yazd, Iran/2009–2013 [12]	Bladder	Cases: 200; Histologically confirmed Controls: 200 healthy neighbors	Questionnaire; Face-to-face interview; Collected data on job titles	Lifetime occupation history: jobs held > 1 year	Age, sex, family history of bladder cancer, chronic urinary tract infections (times per year), kidney and bladder stones, hair dyeing, and educational level	Yes
Ghadimi/Kurdestan, Iran/2012–2015 [13]	Bladder	Cases: 152; Histologically confirmed recruited from cancer registries Controls: 152, Hospital based	Semi-structured questionnaire; Interview; Collected data on job titles, and tasks	Last 20 years jobs	Age, sex, and place of residence, univariable analysis	Yes
Tajvidi/Isfahan, Iran/2001–2010 [14]	Kidney	Cases: 200; kidney cancer cases recruited from cancer registry Controls: 400; healthy population based	Semi-structured questionnaire; Collected data on job title, and self-report of exposures	Not clear	Age, sex	Yes
Aminian/Tehran, Iran/2011–2015 [15]	Non- Hodgkin/Hodgkin’s lymphoma	Cases: 150; male; Histologically confirmed Controls: 150; relative controls (case’s brother or close relative)	Semi-structured questionnaire; Interview; Collected data on job title, duration, exposure to the specific chemicals in each job, history of cancer occurrence in coworkers	Current and former jobs: Jobs held > 1 year	Age, sex	Yes
Zakerinia/Fars, Iran/2007–2008 [16]	Malignant lymphoma	Cases: 200; Histologically confirmed Controls: 200; Hospital based	Semi-structured questionnaire; Face-to-face interview; Collected data on job history, specific exposures for each job, a question on extra jobs in farming including pesticides exposure (herbicides, fungicides, insecticides), reason for exposure, duration	Lifetime occupation history: Jobs held > 1 year	Age, sex, center	Non-Hodgkin lymphoma: Yes Hodgkin lymphoma: No
Amizadeh/Not clear [17]	Head and neck	Cases: 31; Histologically confirmed Controls: 32; Hospital based	Structured questionnaire; Face-to-face interview; Collected data on type of crops, tasks, duration, farming surface size, name of pesticide, frequency of apply per year, and methods, personal protective equipment; biomonitoring (residual pesticide was extracted from adipose tissue)	At least 1 year of agriculture	Age, sex, smoking status	Yes
Hosseini/Tehran, Iran/2002–2005 [18]	Lung	Cases: 242; primary cases; Histologically confirmed Controls: 484; Hospital based (242; Healthy visitors and 242; patients except oncology ward patients)	Structured questionnaire; Face-to-face interview; Collected data on workplace conditions, exposure to suspected occupational lung carcinogens	Not clear	Age, sex, and-place of resident	Yes
Aghilinejad/Tehran, Iran/2014–2015 [19]	Gastrointestinal	Cases: 243; Male; Histologically confirmed Controls: 243; Male; Hospital based (cancer patient other than gastrointestinal cancer)	Questionnaire; Interview; In the method the author stated, “occupational history” but no details provided on the collected data	Childhood until 5 years before cancer diagnosis	Age	Yes
Rafeemanesh/Khorasan-Razavi, Iran/2010–2014 [20]	Breast	Cases: 104; Histologically confirmed (employed women) Controls: 112; Women; Healthy controls (who referred to the health care centers to receive routine examination); Employed women	Questionnaire; Face-to-face Interview; Collected data on recent job title; In the methods “occupational exposures” are stated, with no details provided	Not clear	Not clear	Yes

## Data Availability

All full-texts are available by the first author.

## Supplementary Materials

The following are available online at https://www.mdpi.com/article/10.3390/cancers13143581/s1, Figure S1: Temporal trend of publications on cancer research in occupational settings in Iran, Table S1: Search query used in PubMed to retrieve relevant publications from initiation up to January 2021, Table S2: Search query used in Web of Sciences to retrieve relevant publications from initiation up to January 2021.
