# Supplementary material for: Occupational Exposure to Carcinogens and Occupational Epidemiological Cancer Studies in Iran: A Review
Source: Cancers (Basel). 2021 Jul 16;13(14):3581. doi: 10.3390/cancers13143581 (PMC8305339; doi:10.3390/cancers13143581)
Supplement: Supplementary file 1 [file cancers-13-03581-s001.zip › cancers-1300929-supplementary.pdf]

Supplementary Figures and tables

Supplementary Table S1. Search query used in PubMed to retrieve relevant publications from initiation up to January 2021

	Search	Query	Items found
		**Cancer**	
#1	#1	neoplasms[MeSH Terms]	*2481403*
#2	#2	(cancer* OR carcinoma* OR neoplas* OR tumor* OR tumour* OR malignan*)	*3695197*
		**Occupational exposure**	
#3	#3	Occupational exposure[MeSH Terms]	*59375*
#4	#4	(Occupational hazard* OR workplace hazard* OR hazard*)	*316470*
#5	#5	Industry[MeSH Terms]	*249120*
		**Iran**	
#6	#6	Iran[MeSH Terms]	*26189*
#7	#7	(Iran* OR Tehran* OR Shiraz OR Mazandaran OR Esfahan OR Kerman* OR Yazd OR Arak)	*191212*
#8	#1 OR #2	(neoplasms[MeSH Terms]) OR ((cancer* OR carcinoma* OR neoplas* OR tumor* OR tumour* OR malignan*))	*3872610*
#9	#3 OR #4	(Occupational exposure[MeSH Terms]) OR ((Occupational hazard* OR workplace hazard* OR hazard*))	*368083*
#10	#9 OR #5	(((Occupational exposure[MeSH Terms]) OR ((Occupational hazard* OR workplace hazard* OR hazard*)))) OR Industry[MeSH Terms]	*597564*
#11	#7 OR #8	(iran[MeSH Terms]) OR ((Iran* OR Tehran* OR Shiraz OR Mazandaran OR Esfahan OR Kerman* OR Yazd OR Arak))	*191212*
#14	#9 AND #10 AND #11	(((neoplasms[MeSH Terms] AND (1990:2020[pdat])) OR (cancer* OR carcinoma* OR neoplas* OR tumor* OR tumour* OR malignan* AND (1990:2020[pdat]))) AND (((Occupational exposure[MeSH Terms] AND (1990:2020[pdat])) OR (Occupational hazard* OR workplace hazard* OR hazard* AND (1990:2020[pdat]))) OR (Industry[MeSH Terms] AND (1990:2021[pdat])))) AND ((Iran[MeSH Terms] AND (1990:2020[pdat])) OR (Iran* OR Tehran* OR Shiraz OR Mazandaran OR Esfahan OR Kerman* OR Yazd OR Arak AND (1990:2020[pdat])))	*726*
#15	#14 NOT ((animals[MeSH Terms]) NOT (humans[MeSH Terms])	((((neoplasms[MeSH Terms] AND (1990:2020[pdat])) OR (cancer* OR carcinoma* OR neoplas* OR tumor* OR tumour* OR malignan* AND (1990:2020[pdat]))) AND (((Occupational exposure[MeSH Terms] AND (1990:2020[pdat])) OR (Occupational hazard* OR workplace hazard* OR hazard* AND (1990:2020[pdat]))) OR (Industry[MeSH Terms] AND (1990:2020[pdat])))) AND ((Iran[MeSH Terms] AND (1990:2020[pdat])) OR (Iran* OR Tehran* OR Shiraz OR Mazandaran OR Esfahan OR Kerman* OR	718

		Yazd OR Arak AND (1990:2020[pdat])))) NOT ((animals[MeSH Terms]) NOT (humans[MeSH Terms]) AND (1990:2020[pdat]))	

Supplementary Table S2. Search query used in Web of Sciences to retrieve relevant publications from initiation up to January 2021

	Search	Query	Items found
#1	#1	TS=(cancer* OR carcinoma* OR neoplas* OR tumor* OR tumour* OR malignan*)	3,764615
#2	#2	TS=(Occupational hazard* OR workplace hazard* OR hazard*)	382,311
#3	#3	TS= (industr*)	1,278,655
#4	#4	#3 OR #2	1,634,769
#5	#5	TS=(Iran* OR Tehran* OR Shiraz OR Mazandaran OR Esfahan OR Kerman* OR Yazd OR Arak)	122,013
#6	#6	#5 AND #4 AND #1	391

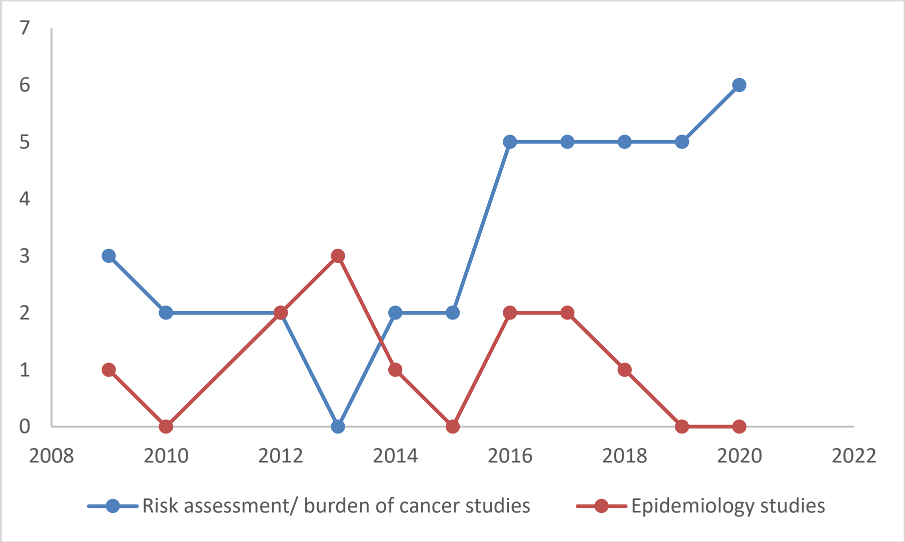

Supplementary Figure S1. Temporal trend of publications on cancer research in occupational settings in Iran
